# Impact of parental rheumatoid arthritis on risk of autism spectrum disorders in offspring: A systematic review and meta-analysis

**DOI:** 10.3389/fmed.2022.1052806

**Published:** 2022-11-10

**Authors:** Cheuk-Kwan Sun, Yu-Shian Cheng, I-Wen Chen, Hsien-Jane Chiu, Weilun Chung, Ruu-Fen Tzang, Hsin-Yi Fan, Chia-Wei Lee, Kuo-Chuan Hung

**Affiliations:** ^1^Department of Emergency Medicine, E-Da Hospital, Kaohsiung City, Taiwan; ^2^School of Medicine for International Students, College of Medicine, I-Shou University, Kaohsiung City, Taiwan; ^3^Department of Psychiatry, Tsyr-Huey Mental Hospital, Kaohsiung Jen-Ai’s Home, Kaohsiung City, Taiwan; ^4^Institute of Biomedical Sciences, National Sun Yat-sen University, Kaohsiung City, Taiwan; ^5^Department of Anesthesiology, Chi Mei Medical Center, Liouying, Tainan City, Taiwan; ^6^Taoyuan Psychiatric Center, Ministry of Health and Welfare, Taoyuan, Taiwan; ^7^Institute of Hospital and Health Care Administration, National Yang-Ming University, Taipei City, Taiwan; ^8^Department of Psychiatry, Mackay Memorial Hospital, Taipei City, Taiwan; ^9^Department of Neurology, Chi-Mei Medical Center, Tainan City, Taiwan; ^10^School of Medicine, College of Medicine, National Sun Yat-sen University, Kaohsiung City, Taiwan; ^11^Department of Anesthesiology, Chi Mei Medical Center, Tainan City, Taiwan

**Keywords:** autism spectrum disorder, rheumatoid arthritis, meta-analysis, offspring, autoimmune diabetes

## Abstract

**Background:**

To investigate the association of risk of offspring autism spectrum disorder (ASD) with both maternal and paternal rheumatoid arthritis (RA).

**Methods:**

The Embase, Medline, Cochrane Library databases were searched for studies that investigated the association of parental RA with risk of offspring ASD. The primary outcome was the associations of maternal/paternal RA with the risk of offspring ASD. Subgroup analyses were conducted based on the timing of maternal RA diagnosis (i.e., before/after childbirth) and geographical location (i.e., Western vs. Asian countries) of studies.

**Results:**

Ten studies published between 2005 and 2022 involving 6,177,650 participants were analyzed. Pooled results revealed a significant association between maternal RA and the risk of ASD (OR = 1.246, *p* < 0.001, 10 studies), while there was no association of paternal RA with the risk of offspring ASD (OR = 1.104, *p* = 0.253, four studies). Subgroup analysis demonstrated no correlation between diagnosis of maternal RA before childbirth and the risk of offspring ASD (OR = 1.449, *p* = 0.192, four studies), while there was a significant association of maternal RA regardless of the timing of diagnosis with the risk of offspring ASD (OR = 1.227, *p* = 0.001, six studies). Subgroup analysis on geographical location showed a significant association of maternal RA with the risk of offspring ASD regardless of the study location (all *p* < 0.05).

**Conclusion:**

Our findings supported an association between maternal RA and an elevated risk of ASD in offspring. However, given the limited numbers of studies investigating the risk of offspring ASD in mothers diagnosed with RA before childbirth, further studies are warranted to elucidate this issue.

**Systematic review registration:**

[www.crd.york.ac.uk/prospero/], identifier [CRD42022358470].

## Introduction

Autism spectrum disorder (ASD), a type of neurodevelopmental disorder, is characterized mainly by deficits in social communication and the presence of repetitive and restrictive behaviors (1). The global prevalence of ASD is estimated to be about 1 in 100 children (2). Children with ASD also frequently present with other comorbid conditions (3) and associated behavioral problems (4) that could pose a significant burden to their family (5) and also society (6). Despite the rise in public awareness of ASD and the increase in prevalence of ASD diagnosis (2), there are still no FDA-approved medications for treating the core symptoms of ASD (7) and early behavioral intervention remains the only currently acceptable strategy for achieving favorable outcome for children diagnosed with ASD (8).

Although available evidence endorses the importance of early treatment for children suffering from ASD (9), early signs of ASD can be difficult to observe especially for those with high-functioning [i.e., normal intelligence quotient (IQ)] autism (10). Therefore, early identification of those at risk of ASD is critical for the prompt implementation of appropriate interventions (11). Despite the complex and possibly multi-factorial etiologies of ASD, most previous research suggested that genes, environment, and gene-environment interactions are the key factors in the pathogenesis of ASD (12). Therefore, the association between several parental illnesses and risks of ASD in their offspring has been an important area in ASD research not only to improve the understanding of the genetic influence on the development of ASD but also to help identify children who may be at an increased risk of the disease (13, 14).

A variety of autoimmune or autoinflammatory diseases in parents, such as inflammatory bowel diseases and inflammatory arthritis, were found to be associated with higher risks of ASD in offspring (13). In particular, rheumatoid arthritis (RA), which is one of the most extensively studied parental autoimmune diseases for its possible link to offspring ASD, has been found to be associated with a higher risk of offspring ASD in many observational studies (15–17). Indeed, a previous meta-analysis also demonstrated an elevated risk of offspring ASD in mothers diagnosed with RA, suggesting a possible link between parental autoimmune diseases and offspring ASD (18). However, that meta-analysis was only able to include five observational studies and did not investigate the correlation between offspring ASD and paternal RA (18). Moreover, some studies only selected mothers who were diagnosed with RA prior to childbirth (19, 20), while others included mothers with their diagnosis of RA being made in any time frame regardless of its relation to childbirth (21, 22). Although maternal RA with an onset prior to childbirth may predispose offspring to the risk of brain maldevelopment through exposing the fetus to an unfavorable pregnancy environment (23–25), the impact of the timing of maternal RA onset with reference to childbirth was not addressed in that meta-analysis (18).

Therefore, to fill this gap in knowledge, the current updated meta-analysis aimed at investigating the association of the risk of offspring ASD with both maternal RA and paternal RA as well as assessing the difference in risk of offspring ASD between mothers who were diagnosis with RA prior to childbirth and those with the diagnosis of RA being made in any time frame.

## Methods

This meta-analysis was reported based on recommendations from the Preferred Reporting Items for Systematic Reviews and Meta-Analyses (PRISMA). The protocol was previously registered in PROSPERO (CRD42022358470).

### Search strategy

Three independent databases including Embase, Medline, and Cochrane Library were searched for the identification of studies that investigated the association of parental RA with the risk of offspring ASD. We also searched Google Scholar and the reference lists of the included studies to retrieve other relevant articles. There were no language and ethnicity restrictions applied for determining study eligibility. The database research was conducted from inception until September 7, 2022. The major terms used for literature search included “Rheumatoid Arthritis” AND “Autism” for literature search. Medical Subject Headings (MeSH), and Embase Subject Headings (Emtree) were also used to facilitate searching. The detailed search strategies for these databases are shown in Supplementary Table 1.

### Studies selection and data extraction

The titles and abstracts of the retrieved articles were independently screened by two authors for eligibility based on the following criteria: (1) observational studies such as cohort or population-based investigations that reported an association between parental RA and risk of offspring ASD; (2) availability of information about odds ratio (OR), relative risk (RR), hazard ratio (HR), or sufficient details (e.g., number of cases) for risk assessment. We excluded the following forms of publications: letters, conference abstracts, meta-analyses, case reports, reviews, and animal experimental studies. For two or more institution-based cohort studies that reported findings on the same or overlapping population, the one with the largest sample size was selected. A third reviewer was consulted for any disagreement.

The following details were extracted from all eligible studies: publication-specific information (e.g., first author’s name, journal, and year of publication) and study characteristics including population size, country, follow-up duration, number of cases, and details for risk calculation [e.g., ORs with corresponding 95% confidence interval (CI)]. If a study reported both unadjusted and adjusted data, we collected the latter for analysis.

### Primary and secondary outcomes

The primary outcomes included the association of maternal RA with the risk of offspring ASD and that of paternal RA with offspring ASD risk regardless of parental age and the timing of RA diagnosis. Two subgroup analyses were performed. The first subgroup investigation was conducted to evaluate the potential impact of pre-childbirth maternal RA (e.g., teratogenicity, pregnancy-related conditions) on the risk of offspring ASD after separating the maternal participants into those diagnosed with RA before childbirth and those whose diagnosis of RA was made without referring to the timing of childbirth, followed by comparing their differences in the risk of offspring ASD relative to that in their respective controls (i.e., mothers without diagnosis of RA). The second subgroup analysis focused on the effect of geographical location (i.e., Western vs. Asian country) on the risk of offspring ASD. The diagnosis of RA and ASD was in accordance with that of each study.

### Quality assessment

Two reviewers independently judged the risk of bias in the retrieved studies based on the Newcastle-Ottawa Scale (NOS), which categorizes the possible sources of bias in a cohort study into eight items pertinent to selection, comparison, and outcome. Disagreements were solved by a third reviewer. A nine-point scale was used to grade the included studies (i.e., low, moderate, and high quality for a score of 0–3, 4–6, and 7–9, respectively).

### Statistical analysis

With the comprehensive Meta-Analysis (CMA) V3 software (Biostat, Englewood, NJ, USA), pooled effect size was estimated using reported patient number or raw data of OR. HR and RR were directly considered to be OR as previously reported (18), taking into account the rarity of ASD. Because this study was conducted based on an observational study design, a random-effects model was used to produce a pooled odds ratio (OR) as in our previous studies (26). Heterogeneity was assessed using the I^2^ statistics in which an I^2^ over 50% denotes substantial heterogeneity (27). Leave-one-out sensitivity analysis was conducted to examine the reliability and the soundness of the available evidence. The potential publication bias for an outcome reported in 10 or more studies was evaluated by visual inspection of a funnel plot and the Egger’s test as previously reported (28, 29). A *p*-value of <0.05 was judged to be statistically significant in the current meta-analysis.

## Results

### Identification of eligible studies

**FIGURE 1:**
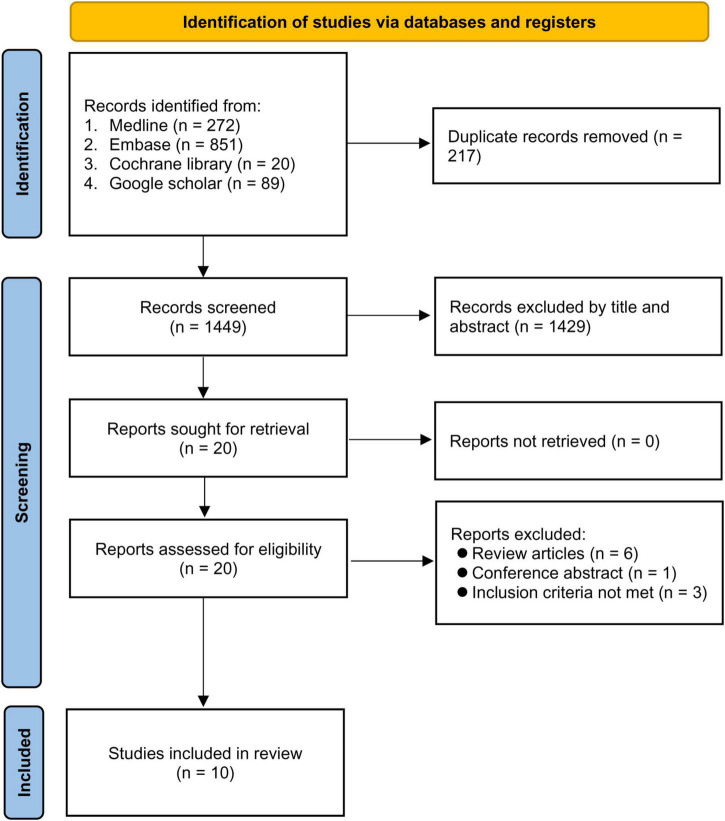
PRISMA flow diagram of study selection.

Our database research retrieved 1,666 records, of which 217 were excluded for being duplicates. Following title and abstract screening, 1,429 records were further removed. Of the remaining 20 articles eligible for full-text reading, 10 were excluded because of being review articles (*n* = 6), conference abstract (*n* = 1), and failure to meet the inclusion criteria (*n* = 3). Finally, 10 studies were eligible for inclusion in this systematic review (15–17, 19–22, 30–32). The flowchart of study selection is demonstrated in Figure 1.

### Study characteristics and risk of bias

Ten studies published between 2005 and 2022 involving 6177650 participants were analyzed (15–17, 19–22, 29, 30, 32). In terms of research designs, six (15–17, 20, 29, 32) and four (19, 21, 22, 30) were cohort and case-control studies, respectively. The method for identification of ASD and RA is shown in Supplementary Table 2. The sample sizes of the studies ranged from 441 to 1,893,244. Of the 10 studies, six documented a relationship between the risk of offspring ASD and mothers diagnosed with RA regardless of timing of maternal RA diagnosis (15–17, 21, 22, 29), four offered data on the risk of offspring ASD associated with the diagnosis of maternal RA prior to childbirth (15, 19, 20, 32), and four provided information about the risk of offspring ASD linked to paternal RA (15, 17, 21, 29). Five studies provided details regarding parental age at the time of offspring ASD diagnosis, which was between 29.05 and 66.6 years. In respect of ethnic backgrounds, six studies were conducted in Western countries (16, 17, 19, 21, 22, 30) and four in Asian countries (15, 20, 29, 32). The mean NOS score of the included studies was 7.7 (SD: 0.48). Detailed information for each included study is provided in Table 1.

**TABLE 1:** Summary of characteristics of included studies in the current meta-analysis (*n* = 10).

References	Design	RA	ASD cases/Total participants	Estimated risks (95% CI)	Parents age (years)	Children age (years)	Female (%) in ASD in offspring	NOS scores	Country
Atladóttir et al. (16)	Cohort study	Mother (b/a childbirth)	3,325/689,196	1.70 (1.07, 2.54)	N/A	N/A	16.15	7	Denmark

Chiu et al. (15)	Cohort study	Father Mother (b/a childbirth)	297/263,791	1.42 (0.7, 2.87) 1.49 (1.01, 2.2)	61.66 61.66	33.95 33.95	19.26	8	Taiwan

Croen et al. (30)	Case control	Mother (2 years before and 2 years after childbirth)	407/2,502	0.858 (0.103, 7.142)	30.01	N/A	18	8	U.S.

Croen et al. (19)	Case control	Mother (before childbirth)	663/1,587	2.09 (0.74, 5.89)	N/A	N/A	17.6	8	U.S.

Lee et al. (20)	Cohort study	Father Mother (before childbirth)	2/4,506	N/A 1.38(0.35, 5.53)	32.47 29.05	4.63	47.6	8	Taiwan

Li et al. (31)	Cohort study	Father Mother (b/a childbirth)	13,885/1,386,260	1.03 (0.82, 1.31) 1.13 (0.94, 1.37)	N/A	9.7	52.2	8	Taiwan

Mouridsen et al. (22)	Case control	Father Mother (b/a childbirth)	111/441	0.627 (0.03, 13.15) 0.49 (0.06, 4.12)	65.58	5.4	26.1	7	Denmark

Rom et al. (17)	Cohort study	Father Mother (b/a childbirth) Mother (after childbirth)	8985/1,917,723	1.33 (0.97–1.82) 1.31 (1.06, 1.63) 1.39 (1.11, 1.75)	N/A	N/A	48.6	8	Denmark

Spann et al. (21)	Case control	Father Mother (b/a childbirth)	4,600/18,400	0.9 (0.6, 1.4) 1.1 (0.8, 1.5)	N/A	8	13.92	7	Finland

Tsai et al. (32)	Cohort study	Mother (before childbirth)	10,631/1,893,244	1.42 (0.60, 3.40)	31.97	6.24	47.8	8	Taiwan

ASD, autism spectrum disorder; b/a, before and after (indicating diagnosis of RA regardless of time of childbirth); CI, confidence interval; N/A, not available; NOS, the Newcastle-Ottawa Scale; RA, rheumatoid arthritis.

### Outcomes

#### Primary outcome: Association between maternal/paternal rheumatoid arthritis and risk of offspring autism spectrum disorder

**FIGURE 2:**
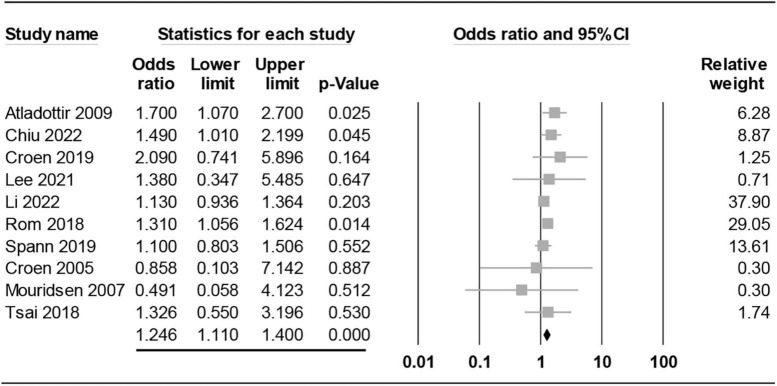
Forest plot showing association of maternal rheumatoid arthritis (RA) with risk of offspring autism spectrum disorder (ASD) regardless of the timing of maternal RA diagnosis. CI, confidence interval.

**FIGURE 3:**
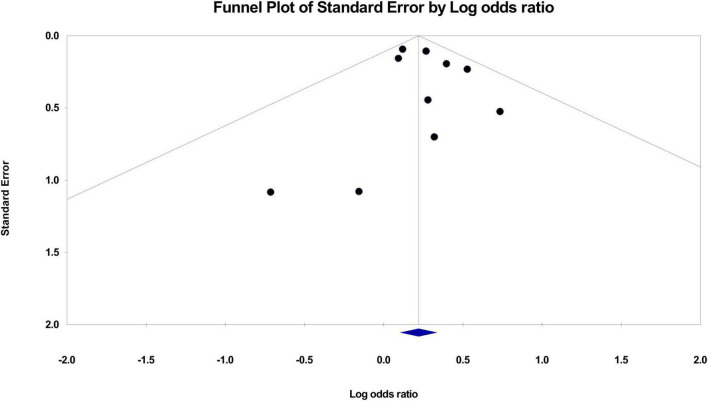
Funnel plot demonstrating risk of publication bias.

Pooled results revealed a significant association between maternal RA and the risk of ASD (OR = 1.246, 95% CI: 1.11 to 1.4, *p* < 0.001; I^2^ = 0%, ten studies) (Figure 2; 15–17, 19–22, 29, 30, 32). Sensitivity analysis demonstrated no significant influence on the results by omitting certain studies. Both funnel plot and Egger’s test indicated a low risk of publication bias (Egger’s test: *p* = 0.595) (Figure 3).

**FIGURE 4:**
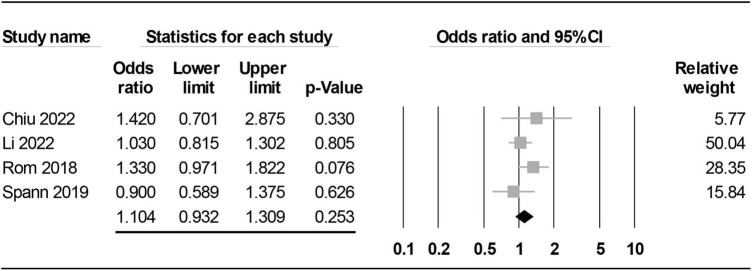
Forest plot showing association of paternal rheumatoid arthritis (RA) with risk of offspring autism spectrum disorder (ASD). CI, confidence interval.

Our analysis demonstrated no impact of paternal RA on the risk of offspring ASD (OR = 1.104, 95% CI: 0.932 to 1.309, *p* = 0.253, I^2^ = 2.127%) (Figure 4; 15, 17, 21, 29). The pooled result was not significantly influenced by removing certain studies on sensitivity analysis.

#### Subgroup analysis: The impact of timing of maternal rheumatoid arthritis diagnosis on risk of offspring autism spectrum disorder

**FIGURE 5:**
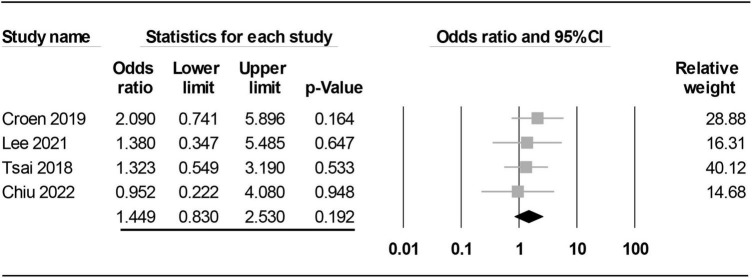
Forest plot showing association between maternal diagnosis of rheumatoid arthritis (RA) before childbirth and risk of offspring autism spectrum disorder (ASD). CI, confidence interval.

**FIGURE 6:**
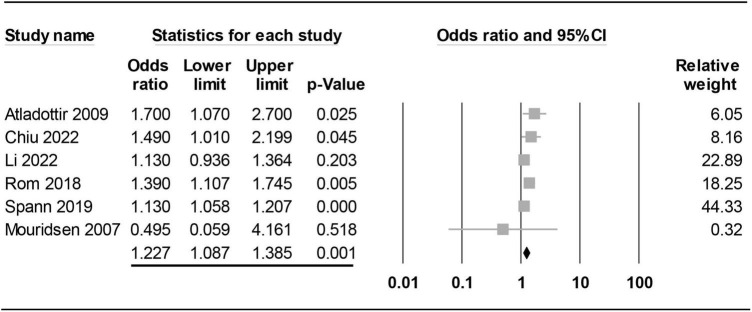
Forest plot showing association of maternal rheumatoid arthritis (RA) regardless of timing of diagnosis (i.e., before or after childbirth) with risk of offspring autism spectrum disorder (ASD). CI, confidence interval.

The impact of timing of maternal RA diagnosis on the risk of offspring ASD is shown in Figures 5, 6. Analysis of the four studies focusing on mothers with pre-childbirth diagnosis of RA (15, 19, 20, 32) demonstrated no significant association between maternal RA and offspring ASD (OR = 1.449, 95% CI: 0.83 to 2.53, *p* = 0.192, I^2^ = 0%, four studies) with consistent findings on sensitivity analysis (Figure 5).

On the other hand, analysis of maternal RA regardless of the timing of diagnosis revealed a significant positive association of maternal RA with the risk of offspring ASD (OR = 1.227, 95% CI: 1.087 to 1.385, *p* = 0.001, I^2^ = 36.911%, six studies) (15–17, 21, 22, 29). The pooled result was not significantly influenced by excluding certain trials on sensitivity analysis (Figure 6).

#### Subgroup analysis: Impact of geographical location on the association between maternal rheumatoid arthritis and risk of offspring autism spectrum disorder

**FIGURE 7:**
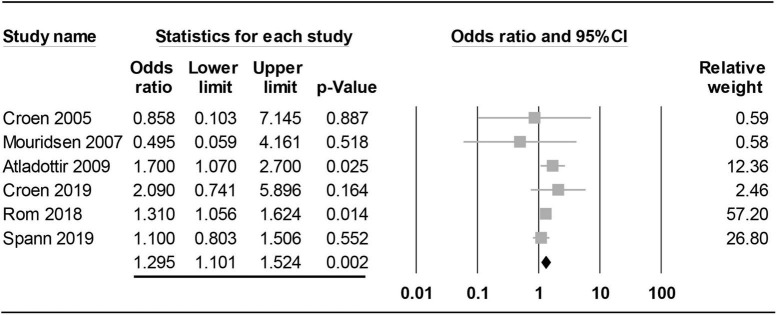
Forest plot showing the correlation between maternal rheumatoid arthritis and risk of offspring autism spectrum disorder in studies from Western countries. CI, confidence interval.

**FIGURE 8:**
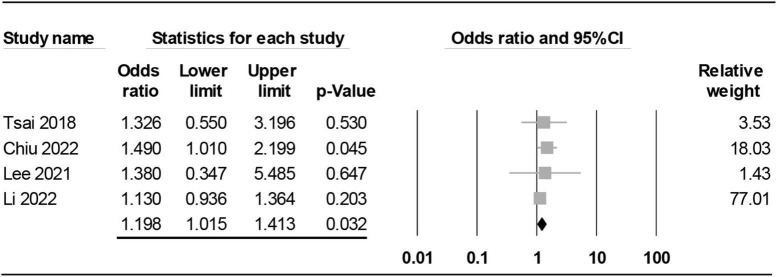
Forest plot demonstrating association of maternal rheumatoid arthritis with risk of offspring autism spectrum disorder in studies from Asian countries. CI, confidence interval.

Findings of subgroup analysis based on geographical location (i.e., Western vs. Asian countries) are shown in Figures 7, 8. Pooled results from studies conducted in Western countries (i.e., European/United States) revealed a significant correlation between maternal RA and offspring RA (OR = 1.295, 95% CI: 1.101 to 1.524, *p* = 0.002, I^2^ = 0%, six studies) (16, 17, 19, 21, 22, 30; Figure 7). Sensitivity analysis demonstrated a loss of significance in this association when one study was removed (17) (OR = 1.283, 95% CI: 0.99 to 1.663, *p* = 0.06).

Focusing on Asian countries, the pooled results also disclosed a significant link between maternal RA and the risk of offspring ASD (OR = 1.198, 95% CI: 1.015 to 1.413, *p* = 0.032, I^2^ = 0%, four studies) (15, 20, 29, 32; Figure 8). Nevertheless, sensitivity analysis showed inconsistent results when one study (15) was excluded (OR = 1.142, 95% CI: 0.951 to 1.37, *p* = 0.155).

## Discussion

Although a previous meta-analysis demonstrated an elevated risk of offspring ASD in mothers suffering from autoimmune diseases, that study only included five studies for RA and investigated only the maternal side (18). Our meta-analysis was updated with 10 studies and was the first to investigate the risk of offspring ASD on the paternal side. To assess the possible influence of pregnancy, we further conducted subgroup analysis on the risk of offspring ASD in mothers diagnosed with RA before childbirth. Albeit statistically non-significant, our subgroup analysis showed a 1.44-fold increase in the risk of offspring ASD in mothers diagnosed with RA before childbirth compared to those without the diagnosis before childbirth, suggesting a potential impact of RA-related maternal changes before childbirth on the risk of ASD in offspring. Whether the absence of statistical significance was attributed to the availability of only four studies for analysis remains unclear. In contrast, comparison of the risk of offspring ASD between a mixed population of mothers diagnosed with RA before and after childbirth and those without RA demonstrated a relatively small 1.25-fold but significant increase in the risk of offspring ASD. On the other hand, the results of our study failed to demonstrate an elevated risk of offspring ASD in fathers diagnosed with RA. Overall, our results not only showed an elevated risk of ASD in offspring associated with maternal RA rather than paternal RA but also suggested an impact of RA-related changes in maternal physical condition (i.e., systemic inflammation or medications) before childbirth on the risk of offspring ASD that warrants further investigations.

Consistent with the results of a previous meta-analysis that demonstrated a 39% increase in risk of ASD in offspring of mothers diagnosed with ASD compared with those without RA (18), our study showed a 25% elevated risk. Compared with that study (18), the inclusion of five more studies (15, 20, 21, 29, 30) in the present meta-analysis provided more robust evidence to support such an elevated risk. Nevertheless, most of the new studies in the current meta-analysis were conducted in the same geographic locations as the previous meta-analysis (18), including Taiwan, U.S., and Denmark with only one new country (Finland) being included in our updated study. Therefore, the similarities in geographic locations and ethnicities of the study populations may partly explain the consistent findings between the previous study (OR: 1.39) (18) and the present meta-analysis (OR: 1.25). Focusing on ethnicity, our subgroup analysis demonstrated comparable increases in risk between Western and Asian countries, suggesting that an elevated risk of ASD in offspring of mothers with RA may be a universal phenomenon. Further studies are required to elucidate the validity of this finding.

There are several possible mechanisms that may explain this elevated risk of offspring ASD in mothers with RA. First, genetic inheritance, which is considered to be one of most important causes of ASD, is supported by several studies that found a shared genetic liability between autoimmune diseases and ASD, such as human leukocyte antigen (HLA) alleles (33–35). Interestingly, this elevated risk in offspring ASD was less obvious or non-significant on paternal side as reported in previous studies (15, 17, 21, 29) and also in our meta-analysis. Therefore, if shared common genetic paths are the main reason for the elevated risk, such a genetic influence seemed only to inherit through the maternal side. However, a previous study investigating a shared risk allele between ASD and RA (i.e., HLA-DR4) during pregnancy ruled out genomic imprinting and mitochondrial allele as the causes of an increased ASD risk in offspring from the mothers (33). Therefore, this elevated risk of offspring ASD on the maternal side may be better explained by changes in maternal condition before childbirth, in which teratogenic effects from shared risk alleles (33) and possible adverse effects from autoimmune mediators (36, 37) or medications (38, 39) during pregnancy may have a role to play in fetal development.

Taking into consideration the importance of environmental factors, especially pregnancy, in the etiology of ASD (36, 37), we further conducted subgroup analysis of studies that focused on mothers diagnosed with RA before childbirth to investigate the effects of pregnancy and other factors before childbirth on the risk of offspring ASD. Although our results showed a 1.44-fold increase in the risk of ASD in offspring among mothers suffering from RA before childbirth compared to those without RA, such an increased risk failed to reach statistical significance (*p* = 0.116) possibly due to the limited numbers of studies (i.e., only four). In contrast, despite the apparently smaller increase in risk from a mixed population comprising women diagnosed with RA before and after childbirth (OR: 1.25), the increase was probably attributable to a larger number of studies (i.e., ten) with a narrower confidence interval.

Because the peak incidence of RA for adults is in their sixties (40) which is long after the fertile period, the genetic influence of maternal RA may be largely diluted if we only included mothers diagnosed with RA prior to childbirth, as a large portion of mothers who carried RA or ASD-related genes were still asymptomatic and mis-classified into the non-RA group for comparison. Indeed, two of our included studies recruiting mother diagnosed with RA regardless of the timing of diagnosis (i.e., before vs. after childbirth) have demonstrated a mean maternal age of around sixties (15, 22), while the mean age of mothers was around thirties in studies that only included those diagnosed with RA before childbirth (20, 30, 32). Therefore, we would expect an underestimation of the risk of offspring ASD in studies that only included mothers with RA before childbirth to assess maternal genetic influence on the risk of ASD in offspring. Nevertheless, a higher risk of offspring RA was observed in our subgroup of mothers suffering from RA prior to childbirth compared to that of a mixed population of mothers with RA diagnosis both before and after childbirth, implicating that pre-childbirth conditions (e.g., pregnancy) may have an important contribution to the risk of ASD in their offspring. Such a proposal was further supported by a study suggesting that risk alleles such as HLA-DR4 may have a teratogenic effect (33). Consistently, the results of other investigations have highlighted that certain conditions during pregnancy such as inflammation or infection (36, 37) may contribute to an increased risk of offspring ASD. For instance, previous animal studies have demonstrated that the elevated levels of cytokines in fetal circulation from *in utero* exposure in mothers with autoimmune diseases were associated with abnormalities in fetal neurodevelopment (36, 37). A clinical correlation between immune mediators and fetal neurodevelopment was further reflected by the finding that the increased level of serum immunoglobulin G (IgG) in women diagnosed with autoimmune diseases (41) could access the fetal compartment during gestation (42) with a higher concentration being found in the circulation of children with autism than that in those with normal development (43). Finally, medications used to relieve pain or control inflammation during pregnancy have also been shown to be associated with an elevated risk of offspring ASD (38, 39), despite the report of a protective effect of NSAID during pregnancy against offspring ASD in one study (44). Therefore, our findings may still implicate the potential impacts of factors before childbirth (e.g., teratogenicity, increased susceptibility during pregnancy) on the risk of offspring ASD. More studies are needed to investigate possible roles of pre-childbirth conditions in mothers suffering RA to gain more insight into possible preventive measures that may be taken to reduce the risk of ASD in offspring.

There are several limitations in the present study. First, although we doubled the number of included studies compared to the previous meta-analysis (18), the additional studies were still conducted in three main geographic locations including Taiwan, Europe, and U.S.; therefore our results may not be extrapolated to populations with other geographic or ethnic backgrounds. Indeed, genetics may be one of most important factors in the etiologies of ASD; a previous study found that the association between certain risk alleles in autoimmune disease and ASD was only observed in Western countries but not in the Chinese population (45), implying a variation in influences of risk alleles among people of different ethnicities. Second, the availability of only four studies for investigating the risk of ASD in offspring of mothers diagnosed with RA before childbirth and the non-significant increase in risk of offspring ASD in this subgroup warrant further studies to verify our findings. Second, because the non-significant increase in the risk of offspring ASD of mothers diagnosed with RA before childbirth was derived from only four studies, further studies are warranted to verify our findings. Third, despite the known associations of other confounders such as an increased body weight of mother (46), medical conditions such as diabetes (47), and certain habits such as smoking (48) with elevated risks of both ASD (49) and offspring RA (48), unavailability of relevant information in most of the included studies precluded the conduction of meaningful subgroup analyses. Fourth, because most of the included studies based their diagnoses of ASD and RA on medical registry or medical records instead of independent validation, a lack of adequate case definition or ascertainment of exposure were the main sources of bias. Fifth, despite our adoption of OR for risk assessment, three studies only provided data on HR (17, 20, 29). Therefore, bias may arise from our using HR to replace OR in our analysis. Finally, since our finding of an absence of association between the risk of offspring ASD and paternal RA were derived from only four studies, this preliminary finding remains to be verified.

## Conclusion

Our finding supported an association between maternal RA and an elevated risk of ASD in offspring. While shared genetic liability may partly explain this correlation, conditions before childbirth may also have an important role to play. However, given the lack of solid case definition and the limited numbers of studies investigating the risk of offspring ASD in mothers diagnosed with RA before childbirth, further studies are warranted to elucidate the maternal effects of RA before childbirth (e.g., pregnancy) on the risk of offspring ASD.

## Data availability statement

The original contributions presented in the study are included in the article/supplementary material, further inquiries can be directed to the corresponding author.

## Author contributions

C-KS and Y-SC: conceptualization and literature search. I-WC and H-JC: methodology. WC and R-FT: trial selection. H-YF: data analysis. H-YF and C-WL: data extraction. C-KS, Y-SC, and K-CH: writing—original draft preparation. K-CH and C-KS: writing—review and editing. All authors had read and agreed to the published version of the manuscript.

## References

[1] Maenner MJ Rice CE Arneson CL Cunniff C Schieve LA Carpenter LA Potential impact of DSM-5 criteria on autism spectrum disorder prevalence estimates. *JAMA Psychiatry.* (2014) 71:292–300. 10.1001/jamapsychiatry.2013.3893 24452504 PMC4041577

[2] Zeidan J Fombonne E Scorah J Ibrahim A Durkin MS Saxena S Global prevalence of autism: a systematic review update. *Autism Res.* (2022) 15:778–90. 35238171 10.1002/aur.2696 PMC9310578

[3] Al-Beltagi M. Autism medical comorbidities. *World J Clin Pediatr.* (2021) 10:15–28. 33972922 10.5409/wjcp.v10.i3.15 PMC8085719

[4] Adams RE Taylor JL Bishop SL. Brief report: ASD-related behavior problems and negative peer experiences among adolescents with ASD in general education settings. *J Autism Dev Disord.* (2020) 50:4548–52. 10.1007/s10803-020-04508-1 32333303

[5] Picardi A Gigantesco A Tarolla E Stoppioni V Cerbo R Cremonte M Parental burden and its correlates in families of children with autism spectrum disorder: a multicentre study with two comparison groups. *Clin Pract Epidemiol Ment Health.* (2018) 14:143–76. 10.2174/1745017901814010143 30158998 PMC6080067

[6] Lavelle TA Weinstein MC Newhouse JP Munir K Kuhlthau KA Prosser LA. Economic burden of childhood autism spectrum disorders. *Pediatrics.* (2014) 133:e520–9. 24515505 10.1542/peds.2013-0763 PMC7034397

[7] Stepanova E Dowling S Phelps M Findling RL. Pharmacotherapy of emotional and behavioral symptoms associated with autism spectrum disorder in children and adolescents. *Dialogues Clin Neurosci.* (2017) 19:395–402. 29398934 10.31887/DCNS.2017.19.4/rfindling PMC5789216

[8] Pasco G. The value of early intervention for children with autism. *Paediatr Child Health.* (2018) 28:364–7.

[9] Warren Z McPheeters M Sathe N Foss-Feig J Glasser A Veenstra-VanderWeele J. A systematic review of early intensive intervention for autism spectrum disorders. *Pediatrics.* (2011) 127:e1303–11. 21464190 10.1542/peds.2011-0426

[10] Hus Y Segal O. Challenges surrounding the diagnosis of autism in children. *Neuropsychiatr Dis Treat.* (2021) 17:3509–29. 34898983 10.2147/NDT.S282569 PMC8654688

[11] Johnson C Myers S. Identification and evaluation of children with autism spectrum disorders. *Pediatrics.* (2007) 120:1183–215. 17967920 10.1542/peds.2007-2361

[12] Chaste P Leboyer M. Autism risk factors: genes, environment, and gene-environment interactions. *Dialogues Clin Neurosci.* (2012) 14:281–92. 23226953 10.31887/DCNS.2012.14.3/pchaste PMC3513682

[13] Ellul P Acquaviva E Peyre H Rosenzwajg M Gressens P Klatzmann D Parental autoimmune and autoinflammatory disorders as multiple risk factors for common neurodevelopmental disorders in offspring: a systematic review and meta-analysis. *Trans Psychiatry.* (2022) 12:112. 10.1038/s41398-022-01843-y 35304436 PMC8933391

[14] Ayano G Maravilla J Alati R. Risk of autistic spectrum disorder in offspring with parental mood disorders: a systematic review and meta-analysis. *J Affect Disord.* (2019) 248:185–97. 10.1016/j.jad.2019.01.038 30739049

[15] Chiu HJ Sun CK Tsai SJ Bai YM Hung KC Hsu JW A nationwide study of the risks of major mental disorders among the offspring of parents with rheumatoid arthritis. *Sci Rep.* (2022) 12:4962. 10.1038/s41598-022-08834-5 35322089 PMC8943140

[16] Atladóttir HO Pedersen MG Thorsen P Mortensen PB Deleuran B Eaton WW Association of family history of autoimmune diseases and autism spectrum disorders. *Pediatrics.* (2009) 124:687–94. 19581261 10.1542/peds.2008-2445

[17] Rom AL Wu CS Olsen J Jawaheer D Hetland ML Mørch LS. Parental rheumatoid arthritis and autism spectrum disorders in offspring: a Danish nationwide cohort study. *J Am Acad Child Adolesc Psychiatry.* (2018) 57:28–32.e1. 10.1016/j.jaac.2017.10.002 29301665

[18] Zhu Z Tang S Deng X Wang Y. Maternal systemic lupus erythematosus, rheumatoid arthritis, and risk for autism spectrum disorders in offspring: a meta-analysis. *J Autism Dev Disord.* (2020) 50:2852–9. 10.1007/s10803-020-04400-y 32034648

[19] Croen LA Qian Y Ashwood P Daniels JL Fallin D Schendel D Family history of immune conditions and autism spectrum and developmental disorders: findings from the study to explore early development. *Autism Res.* (2019) 12:123–35. 10.1002/aur.1979 30095240 PMC6467644

[20] Lee H Hsu JW Tsai SJ Huang KL Bai YM Su TP Risk of attention deficit hyperactivity and autism spectrum disorders among the children of parents with autoimmune diseases: a nationwide birth cohort study. *Eur Child Adolesc Psychiatry.* (2021). [Epub ahead of print]. 10.1007/s00787-021-01860-0 34387733

[21] Spann MN Timonen-Soivio L Suominen A Cheslack-Postava K McKeague IW Sourander A Proband and familial autoimmune diseases are associated with proband diagnosis of autism spectrum disorders. *J Am Acad Child Adoles Psychiatry.* (2019) 58:496–505. 10.1016/j.jaac.2018.09.444 PMC6631342 30975444

[22] Mouridsen SE Rich B Isager T Nedergaard NJ. Autoimmune diseases in parents of children with infantile autism: a case-control study. *Dev Med Child Neurol.* (2007) 49:429–32. 17518928 10.1111/j.1469-8749.2007.00429.x

[23] Atladóttir H Henriksen TB Schendel DE Parner ET. Autism after infection, febrile episodes, and antibiotic use during pregnancy: an exploratory study. *Pediatrics.* (2012) 130:e1447–54. 10.1542/peds.2012-1107 23147969 PMC4451062

[24] Wu WL Hsiao EY Yan Z Mazmanian SK Patterson PH. The placental interleukin-6 signaling controls fetal brain development and behavior. *Brain Behav Immun.* (2017) 62:11–23. 10.1016/j.bbi.2016.11.007 27838335 PMC5373986

[25] Rudolph MD Graham AM Feczko E Miranda-Dominguez O Rasmussen JM Nardos R Maternal IL-6 during pregnancy can be estimated from newborn brain connectivity and predicts future working memory in offspring. *Nat Neurosci.* (2018) 21:765–72. 10.1038/s41593-018-0128-y 29632361 PMC5920734

[26] Hung KC Wang LK Lin YT Yu CH Chang CY Sun CK Association of preoperative vitamin D deficiency with the risk of postoperative delirium and cognitive dysfunction: a meta-analysis. *J Clin Anesthes.* (2022) 79:110681. 10.1016/j.jclinane.2022.110681 35255352

[27] Hung KC Chu CC Hsing CH Chang YP Li YY Liu WC Association between perioperative intravenous lidocaine and subjective quality of recovery: a meta-analysis of randomized controlled trials. *J Clin Anesthes.* (2021) 75:110521. 10.1016/j.jclinane.2021.110521 34547603

[28] Hung K-C Ko C-C Wang L-K Liu P-H Chen I-W Huang Y-T Association of prognostic nutritional index with severity and mortality of hospitalized patients with covid-19: a systematic review and meta-analysis. *Diagnostics.* (2022) 12:1515. 10.3390/diagnostics12071515 PMC9322949 35885421

[29] Hung K-C Huang Y-T Chang Y-J Yu C-H Wang L-K Wu C-Y Association between fibrinogen-to-albumin ratio and prognosis of hospitalized patients with covid-19: a systematic review and meta-analysis. *Diagnostics.* (2022) 12:1678. 10.3390/diagnostics12071678 35885582 PMC9317445

[30] Croen LA Grether JK Yoshida CK Odouli R Van de Water J. Maternal autoimmune diseases, asthma and allergies, and childhood autism spectrum disorders: a case-control study. *Arch Pediatr Adoles Med.* (2005) 159:151–7. 10.1001/archpedi.159.2.151 15699309

[31] Li DJ Tsai CS Hsiao RC Chen YL Yen CF. Associations between allergic and autoimmune diseases with autism spectrum disorder and attention-deficit/hyperactivity disorder within families: a population-based cohort study. *Int J Environ Res Public Health.* (2022) 19:4503. 10.3390/ijerph19084503 35457368 PMC9025211

[32] Tsai PH Yu KH Chou IJ Luo SF Tseng WY Huang LH Risk of autism spectrum disorder in children born to mothers with systemic lupus erythematosus and rheumatoid arthritis in Taiwan. *Joint Bone Spine.* (2018) 85:599–603. 10.1016/j.jbspin.2017.11.005 29183859

[33] Johnson WG Buyske S Mars AE Sreenath M Stenroos ES Williams TA HLA-DR4 as a risk allele for autism acting in mothers of probands possibly during pregnancy. *Arch Pediatr Adoles Med.* (2009) 163:542–6. 10.1001/archpediatrics.2009.74 19487610

[34] Mostafa GA Shehab AA. The link of C4B null allele to autism and to a family history of autoimmunity in Egyptian autistic children. *J Neuroimmunol.* (2010) 223:115–9. 10.1016/j.jneuroim.2010.03.025 20452682

[35] Gesundheit B Rosenzweig JP Naor D Lerer B Zachor DA Procházka V Immunological and autoimmune considerations of Autism Spectrum Disorders. *J Autoimmun.* (2013) 44:1–7. 23867105 10.1016/j.jaut.2013.05.005

[36] Brimberg L Sadiq A Gregersen PK Diamond B. Brain-reactive IgG correlates with autoimmunity in mothers of a child with an autism spectrum disorder. *Mol Psychiatry.* (2013) 18:1171–7. 10.1038/mp.2013.101 23958959

[37] Smith SE Li J Garbett K Mirnics K Patterson PH. Maternal immune activation alters fetal brain development through interleukin-6. *J Neurosci.* (2007) 27:10695–702. 17913903 10.1523/JNEUROSCI.2178-07.2007 PMC2387067

[38] Ji Y Azuine RE Zhang Y Hou W Hong X Wang G Association of cord plasma biomarkers of in utero acetaminophen exposure with risk of attention-deficit/hyperactivity disorder and autism spectrum disorder in childhood. *JAMA Psychiatry.* (2020) 77:180–9. 10.1001/jamapsychiatry.2019.3259 31664451 PMC6822099

[39] Rifas-Shiman SL Cardenas A Hivert MF Tiemeier H Bertoldi AD Oken E. Associations of prenatal or infant exposure to acetaminophen or ibuprofen with mid-childhood executive function and behaviour. *Paediatr Perin Epidemiol.* (2020) 34:287–98. 10.1111/ppe.12596 PMC7170759 31637744

[40] Myasoedova E Crowson CS Kremers HM Therneau TM Gabriel SE. Is the incidence of rheumatoid arthritis rising?: results from Olmsted County, Minnesota, 1955-2007. *Arthritis Rheumatism.* (2010) 62:1576–82. 10.1002/art.27425 20191579 PMC2929692

[41] Aho K Heliövaara M Knekt P Reunanen A Aromaa A Leino A Serum immunoglobulins and the risk of rheumatoid arthritis. *Ann Rheumatic Dis.* (1997) 56:351–6. 10.1136/ard.56.6.351 PMC1752401 9227163

[42] Fox-Edmiston E Van de Water J. Maternal anti-fetal brain Igg autoantibodies and autism spectrum disorder: current knowledge and its implications for potential therapeutics. *CNS Drugs.* (2015) 29:715–24. 10.1007/s40263-015-0279-2 26369920 PMC4605883

[43] Enstrom A Krakowiak P Onore C Pessah IN Hertz-Picciotto I Hansen RL Increased IgG4 levels in children with autism disorder. *Brain Behav Immun.* (2009) 23:389–95. 19136055 10.1016/j.bbi.2008.12.005 PMC2696343

[44] West R Tseng T Adams ML Rich W Hill E Tillman D Autism and gestational exposures to nonsteroidal anti-inflammatory drugs (NSAIDs) and acetaminophen. *Pharm Sci.* (2013) 240.

[45] Chien YL Wu YY Chen CH Gau SS Huang YS Chien WH Association of HLA-DRB1 alleles and neuropsychological function in autism. *Psychiatr Genet.* (2012) 22:46–9. 10.1097/YPG.0b013e32834915ae 21716163

[46] Lauritsen MB Jørgensen M Madsen KM Lemcke S Toft S Grove J Validity of childhood autism in the Danish Psychiatric Central Register: findings from a cohort sample born 1990-1999. *J Autism Dev Disord.* (2010) 40:139–48. 10.1007/s10803-009-0818-0 19728067

[47] Xu G Jing J Bowers K Liu B Bao W. Maternal diabetes and the risk of autism spectrum disorders in the offspring: a systematic review and meta-analysis. *J Autism Dev Disord.* (2014) 44:766–75. 24057131 10.1007/s10803-013-1928-2 PMC4181720

[48] Modabbernia A Velthorst E Reichenberg A. Environmental risk factors for autism: an evidence-based review of systematic reviews and meta-analyses. *Mol Autism.* (2017) 8:13. 10.1186/s13229-017-0121-4 28331572 PMC5356236

[49] Chang K Yang SM Kim SH Han KH Park SJ Shin JI. Smoking and rheumatoid arthritis. *Int J Mol Sci.* (2014) 15:22279–95. 25479074 10.3390/ijms151222279 PMC4284707

