# Supplementary material for: Impact of parental rheumatoid arthritis on risk of autism spectrum disorders in offspring: A systematic review and meta-analysis
Source: Front Med (Lausanne). 2022 Nov 10;9:1052806. doi: 10.3389/fmed.2022.1052806 (PMC9687371; doi:10.3389/fmed.2022.1052806)
Supplement: Supplementary file 1 [file Data_Sheet_1.docx]

**Supplemental Table 1. Search strategies for databases**

| Database | # | Search syntax |
| --- | --- | --- |
| **MEDLINE (Ovid)** | 1 | ("Rheumatoid Arthritis" OR "RA").mp |
|  | 2 | exp "Arthritis, Rheumatoid"/ |
|  | 3 | ("Autism Spectrum Disorder*" OR "Autistic Spectrum Disorder*" OR "ASD" OR "Autism" OR "autistic disorder" OR "Kanner's syndrome" OR "Asperger’s Syndrome" OR "pervasive developmental disorder").mp |
|  | 4 | exp "Autism Spectrum Disorder"/ |
|  | 5 | (#1 or #2) and (#3 or #4) |
| **Cochrane**  **CENTRAL** | 1 | ("Rheumatoid Arthritis" OR "RA"):ti,ab,kw |
|  | 2 | [mh "Arthritis, Rheumatoid"] |
|  | 3 | ("Autism Spectrum Disorder*" OR "Autistic Spectrum Disorder*" OR "ASD" OR "Autism" OR "autistic disorder" OR "Kanner's syndrome" OR "Asperger’s Syndrome" OR "pervasive developmental disorder"):ti,ab,kw |
|  | 4 | [mh "Autism Spectrum Disorder"] |
|  | 5 | (#1 or #2) and (#3 or #4) |
| **Embase** | 1 | ("Rheumatoid Arthritis" OR "RA")::ti,ab,kw,de |
|  | 2 | "Arthritis, Rheumatoid"/exp |
|  | 3 | ("Autism Spectrum Disorder*" OR "Autistic Spectrum Disorder*" OR "ASD" OR "Autism" OR "autistic disorder" OR "Kanner's syndrome" OR "Asperger’s Syndrome" OR "pervasive developmental disorder"):ti,ab,kw,de |
|  | 4 | "Autism Spectrum Disorder"/exp |
|  | 5 | (#1 or #2) and (#3 or #4) |

**Supplemental Table 2.** Method for identification of ASD and RA cases

| Identification of ASD cases | Identification of RA diagnosis in parents |
| --- | --- |
| Medical records in registry, ICD-10 | Medical records in registry, ICD-10 |
| Health Insurance database, ICD-9 | Health Insurance database, ICD-9 |
| KPMCP facility, ICD-9 | KPMCP facility, ICD-9 |
| from SEED study, by SCQ, ADOS and ADI-R | Self-report or medical records |
| Health Insurance database, ICD-9 | Health Insurance database, ICD-9 |
| Health Insurance database, ICD-9 and 10 | Health Insurance database, ICD-9 and 10 |
| Medical records, ICD-8/ICD-10 | Medical records in registry, ICD-8/ICD-10 |
| Danish National Hospital Registry, ICD-8/ ICD-10 | Danish National Hospital Registry, ICD-8/ ICD-10 |
| Finnish national registries (FHDR, FMBR & FCPR, ICD-9/ ICD-10 | Finnish national registries (FHDR, FMBR & FCPR, ICD-9/ ICD-10 |
| Health Insurance database, ICD-9 | Health Insurance database |

ASD, Autism spectrum disorder; RA, rheumatoid arthritis SCQ, Social Communication Questionnaire; ADOS, Autism Diagnostic Observation Schedule; ADI-R, Autism Diagnostic Interview-Revised; FMBR, Finnish Medical Birth Register; ICD, International Classification of Diseases; FHDR, Finnish Hospital Discharge Register; FCPR, Finnish Central Population Register; SEED Study to Explore Early Development; KPMCP, Kaiser Permanente Medical Care Program.
